# Network Analysis of the Organic Chemistry in Patents, Literature, and Pharmaceutical Industry

**DOI:** 10.1002/minf.202500011

**Published:** 2025-07-18

**Authors:** Emma Svensson, Emma Granqvist, Tomas Bastys, Christos Kannas, Mikhail Kabeshov, Samuel Genheden, Ola Engkvist, Thierry Kogej

**Affiliations:** ^1^ Molecular AI, Discovery Sciences, R&D AstraZeneca Gothenburg Sweden; ^2^ ELLIS Unit Linz & Institute for Machine Learning Johannes Kepler University Linz Linz Austria; ^3^ Department of Computer Science and Engineering Chalmers University of Technology and University of Gothenburg Gothenburg Sweden

**Keywords:** chemical reactions, graph analysis, knowledge graph, organic chemistry

## Abstract

Chemical reactions can be connected in large networks such as knowledge graphs. In this way, prior work has been able to draw meaningful conclusions about the properties and structures involved in organic chemistry reactions. However, the research has focused on public sources of organic synthesis that might lack the intricate details of the synthetic routes used in in‐house drug discovery. In this work, previous analyses are expanded to also include an in‐house electronic lab notebook (ELN) source, such that we can compare it to knowledge graphs that were constructed from US Patent and Trademark Office (USPTO) and Reaxys. We found that the Reaxys knowledge graph is the most interconnected and has the largest proportion of nodes belonging to the core, whereas the USPTO is much less connected and only has a small core. The ELN knowledge graph falls between these extremes in connectivity and it does not have any core. The hub molecules of ELN and USPTO are most similar, primarily represented by small, organic building blocks. We hypothesize that these differences can be attributed to the different origins of the data in the three sources. We discuss what impact this might have on synthesis prediction modelling.

## Graphical abstract

Knowledge graphs built on AstraZeneca electronic lab notebook, US Patent and Trademark Office (USPTO) and Reaxys chemical reaction data are compared using various knowledge graph metrics.

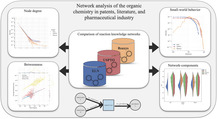

## 1 Introduction

The synthesis of chemical matter is at the heart of organic chemistry and is an essential activity in the pharmaceutical industry [1, 2]. Past experiments form a vast collection of knowledge that could be harnessed for making decisions on future experiments. Currently, most of the knowledge about chemical reactions is stored and accessed through large databases such as [3] and [4], containing manually curated records from various sources. Furthermore, a large knowledge base is embedded in patents, mined into machine‐readable records [5]. Finally, there is a large quantity of data in (electronic) lab notebooks in both academia and industry that in most cases have not been analysed. Common to all these sources of experimental data is that they offer the potential for scientists to query individual reactions. This approach has been widely practiced by the synthesis and medicinal chemistry community during multistep and individual synthesis planning (ref to reaxys/scifinder). More recently, it has been proposed that additional knowledge could be extracted if all of these experimental records were considered jointly, i.e. if they were considered as a network of organic chemistry [6].

Network theory [7] offers an approach to analyse a network of organic chemistry and has been explored in some publications before. Bishop, Klajn & Grzybowski [6] analysed a few million reactions from the Beilstein database (a precursor to Reaxys) and concluded that the network of organic chemistry is a well‐defined architecture with an inherent modular structure. They furthermore explored the evolution of this network over time and found that it evolves through a mechanism called preferential attachment [8]. This means that nodes, i.e. molecules, form new connections at a rate proportional to their current number of connections. As a result, well‐connected nodes become even more connected over time. Preferential attachment can be inferred from the scale‐free property of the network. In a scale‐free network, the node connectivity distribution follows a power law, where a small proportion of the nodes have a higher number of connections than the average while at the same time, the majority of nodes have few connections [9]. Scale‐free networks have been extensively researched in many areas [10].

The seminal work from [6] was followed up by [11] on a much larger dataset extracted from the Reaxys database. They confirmed the scale‐free property of the network and also provided additional analyses based on network theory. Furthermore, these two studies differ in how they construct the network. Bishop, Klajn & Grzybowski [6] built a bipartite graph, where the network is a directed graph constituting two sets of nodes representing either molecules or reactions. In contrast [11], built a network where molecules are nodes, and the edges represent the reactions. Because many reactions have more than one reactant, more than one edge can represent each reaction. An alternative approach was introduced by [12], to use a hypergraph representation of the network. In their approach, the molecular nodes are connected via hyper‐edges that represent reactions. The hyper‐edges can connect to multiple nodes, thus embedding a relationship between the reactants and products in a given reaction. The hypergraph approach was compared to the directed graph approach on a dataset from the US Patent and Trademark Office (USPTO) [5].

It is worth mentioning that chemical reaction knowledge graphs, compared to traditional frequency‐based statistical analysis, can enable machine learning models to leverage not only direct reactant–product relationships but also the extended paths of connection within the reaction network. By incorporating such structured information, machine learning algorithms can improve the predictive accuracy of a specific modelling task. For instance [13], developed a self‐supervised contrastive learning framework that integrates a knowledge graph into molecular representation learning. Their approach allowed models to capture reaction‐based molecular relationships more effectively, leading to improved modelling performance in tasks such as reaction classification, product prediction, and yield prediction. Similarly [14], explored how a knowledge graph could expand the accessible chemical space by encoding transformations between reactants and products. Their study demonstrated that by utilizing a reaction knowledge graph, it was possible to identify new viable reactions and generate libraries of novel compounds that would have been difficult to infer using other methods.

Considering the various sources of chemical reactions, such as commercial databases, patents, and not the least electronic lab notebooks—it would be of interest to compare the knowledge content in different sources. However, previous studies have primarily focused on a single source of reaction data that also might not be representative of the experiments performed in the pharmaceutical industry. In this study, we use a similar methodology as the aforementioned studies [6, 11] to analyse different networks of organic chemistry constructed from three different reaction datasets: 1) Reaxys, 2) the US Patent and Trademark Office (USPTO), and 3) an in‐house electronic lab notebook (ELN). Our primary aim is to provide a comprehensive comparison and to compose hypotheses on the observed differences.

## 2 Methods

### 2.1 Datasets and Knowledge Graphs

We constructed reaction knowledge graphs from three data sources: 1) reactions mined from USPTO, 2) Reaxys, and 3) an in‐house ELN. The source of the USPTO dataset was the original data deposited on Figshare, and we reassigned the atom mapping using NameRXN from [15] whenever possible. For Reaxys and ELN, we extracted data from an internal database that combines several reaction sources into a common structure. The Reaxys data analysed here contains all reactions up to the end of 2020. The ELN data contains a set of reactions up to June 2023 with a recorded yield of 5% or more. Atom mapping was assigned with NameRXN whenever possible, and with Pipeline Pilot [16] otherwise, because being rule‐based, NameRXN is superior to the greedy algorithm in Pipeline Pilot.

We used an identical extract‐transform‐load (ETL) pipeline for all three data sources to add the reactions to a knowledge graph. We identified reactants as the components to the left or above the reaction arrow that share at least one atom mapping number with the product, and these were moved to the left of the reaction arrow. All other components were assigned as reagents and kept above the reaction arrow. Then, we neutralized the reactants and products using RDKit uncharger [17]. All reactions that did not fulfil the following criteria were removed: 1) being single step, 2) having a single product, 3) including at least one reactant, 4) having a product different from the reactant, and 5) containing no dummy atoms.

**FIGURE 1:**
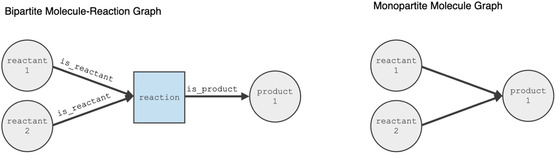
Comparison of bipartite (left) and monopartite (right) graph representations of a bimolecular reaction.

From the resulting reaction data, we constructed a bipartite graph similar to the one proposed by [18] for each data source, with nodes representing either molecules or reactions. A reaction in this context is defined without consideration of the reagents, i.e. reactions that only differ in the reaction condition were grouped into a single node. A molecule and a reaction node were connected with an edge indicating whether the molecule was a reactant or product in the reaction. To explore other aspects of the networks, we additionally created two monopartite graphs containing only molecule or reaction nodes. In the molecule graph, reactants and product molecule nodes were connected with an edge if the reactant and product were part of a reaction. Similarly, in the reaction graph, reaction nodes were connected if they shared a common reactant or product. Figure 1 illustrates the difference between the bipartite and monopartite graph for a single bimolecular reaction with a single product.

### 2.2 Graph Analysis

We performed several analyses on the graphs described above following the methods detailed by [11]. First, we evaluated the scale‐free properties of each graph by calculating the in‐ and out‐degree for each node and analysing the resulting degree distributions. Specifically, each empirical distribution's fit to a power law distribution was statistically compared to its fit to other distributions, including log‐normal, positive log‐normal, truncated power law, exponential, and stretched exponential distributions. Furthermore, we identified key components of the graphs, such as islands, periphery, core, and hubs. We also calculated the average shortest path lengths and measured the hierarchy of the network.

The graph analysis was performed using the graph‐tool Python package [19] with implementations of an extensive set of graph algorithms. We primarily used the available algorithms for node‐degree, shortest path lengths, clustering, and betweenness. Additionally, these algorithms are prepared for parallelization, which makes it convenient to handle large datasets such as the ones analysed in this work. For the evaluation of the power law distribution, we used the powerlaw package proposed by [20] which includes statistical tests comparing the empirical distribution to power law and other relevant theoretical distributions.

Finally, a variety of molecular properties were calculated using RDKit [17] for all molecular compounds included in the reactions. We considered properties related to the complexity and drug‐likeliness of the compounds. The complexity was assessed by the molecular weight, number of heavy atoms, number of rings, number of chiral atoms, and the fraction of carbon atoms that are sp3 hybridized, i.e. Fsp3. The Quantitative Estimator of Drug‐likeliness (QED) score was used to estimate drug‐likeliness [21]. A QED score close to 1 indicates that a compound is drug‐like, while a score close to 0 indicates that it is not. The averages of these properties were then compared between graphs as well as between different sub‐structures of each graph.

## 3 Results

The following section presents the findings from our analysis of several chemistry reaction knowledge graphs, constructed from a set of reactions in an in‐house ELN, USPTO, and Reaxys respectively. Additionally, each bipartite graph has been complemented with two monopartite graphs, which contain only molecule nodes versus only reaction nodes. Table 1 presents the number of nodes (reactions and molecules) and edges (reactants and products) across three datasets: ELN, USPTO, and Reaxys. Among them, ELN is the smallest, while Reaxys is significantly larger, containing more than 10 times the number of reactions and molecules compared to USPTO. The number of edges follows a similar trend. Reaxys has nearly 24 times more edges than ELN and about 15 times more than USPTO. The number of reactants in Reaxys (24M) alone surpasses the total number of those in both USPTO (1.6M) and ELN (2.6M) combined, highlighting the much richer reaction data available in Reaxys. A useful way to measure graph connectivity is the edge‐to‐node ratio. This ratio is 1.486 for Reaxys, 1.304 for ELN, and 1.184 for USPTO, indicating that Reaxys is the most densely connected graph, while USPTO is the sparsest. A higher ratio suggests that each molecule or reaction is involved in more connections, which may indicate a broader coverage of reaction pathways in Reaxys. The large size of Reaxys is likely due to its extensive coverage of both patent and non‐patent literature, as well as manually curated experimental data. In contrast, USPTO focuses primarily on patent reactions, which are more selective, and ELN represents a collection of proprietary or experimental reaction data, making it the smallest dataset.

**TABLE 1:** Graph statistics. Counts of nodes and edges in each graph.

	ELN	USPTO	Reaxys
Nodes	
Reactions	556,470	934,948	13,120,771
Molecules	636,361	1,224,059	11,851,702
Total	1,192,831	2,159,007	24,972,473
Edges	
Products	556,470	934,948	13,120,771
Reactants	998,627	1,621,340	23,991,669
Total	1,555,097	2,556,288	37,112,440

### 3.1 The Scale‐Free Property

**FIGURE 2:**
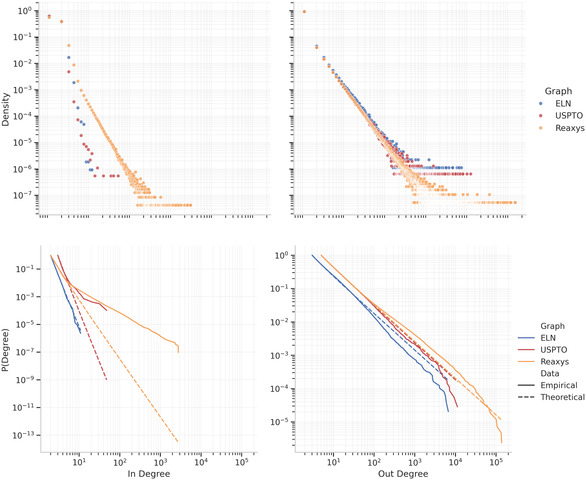
Distribution of the degree distributions. (Upper) Plot of the distributions of the in and out‐degree. (Lower) Cumulative distribution for the empirical in‐ and out‐degree distribution as solid lines, compared to the fitted theoretical power law distributions as dashed lines.

The degree distributions of the bipartite graphs are shown as normalized histograms in the upper row of Figure 2. It can be seen that many nodes have only a few connections while fewer ones are highly connected, similarly to what was previously reported by [11, 12] although those graph was wired differently than our bi‐partite graphs. Complementary, Table S1 presents the average degree in each version of the graphs, but as [11] points out, the average degree is a poor metric of the general graph's properties.

To gain a more in‐depth understanding of the graph's respective attachment mechanisms, each degree distribution is also fitted to a power law. Prior work has shown that similar chemical networks follow a power law distribution [20], which indicates a preferential attachment style known as the scale‐free property. The scale‐invariant relation is described as,

(1) $$P(k)\propto k^{-\gamma }$$

where P(k) is the probability to observe a node with degree k and γ is the power law exponent. The scale invariant property means that the distribution retains its features regardless of the scale of the variable. A power law is a heavy‐tailed distribution, meaning that the tail of the distribution is not exponentially bounded [20].

The empirical distributions of the Complement Cumulative Distribution Function (CCDF) are shown next to the respective, best‐fitted theoretical power laws in the bottom row of Figure 2. While [11] used a fixed start value of degree $k_{\text{min}}=1$ to evaluate the power law fit, we adopt the best possible fit by letting the algorithm choose the most optimal $k_{\text{min}}$. The resulting values on $k_{\text{min}}$ for each graph, as well as the fitted parameter γ are all provided in Table 2.

**TABLE 2:** Power law fit. Estimated parameters for fitting the degree distributions to a power law, presenting the obtained power law exponents, *γ*, as well as the optimal minimum degree used, $k_{\text{min}}$.

	ELN		USPTO		Reaxys	
	γ	$k_{\text{min}}$	γ	$k_{\text{min}}$	γ	$k_{\text{min}}$
In	7.77	2	8.16	3	5.19	2
Out	2.10	3	2.12	5	2.10	5

Note that the optimal $k_{\text{min}}$ values are between 2 and 5 for all graphs and degree types. Despite using a few degrees higher than previous work, we observe the same results for all graphs in terms of out‐degree, i.e. $\gamma_{\text{out}}\approx 2.1$ [11, 18]. However, $\gamma_{\text{in}}$ ranges between 5.2 for Reaxys and 8.2 for USPTO, which is larger than the previously reported $\gamma_{\text{in}}\approx 3$ [11, 12]. The difference between our values and those previously reported might be due to the difference in how the datasets were processed or how the networks were wired. Furthermore, from the CCDFs, it is clear that the distributions fit the power law well in terms of out‐degree but not necessarily for all graphs in terms of in‐degree. ELN is the only graph for which the CCDF fits the power law well in terms of in‐degree, although with a steeper slope than previously seen. For the remaining graphs, we need further analysis to examine the scale‐free properties of the in‐degree.

Additional details are provided in the Supporting Information, where Table S2 presents likelihood‐ratio tests for comparing how well the degree distributions fit a power law compared to other distributions. Based on these results, we can rule out that either of the degree distributions are best described by exponential, stretched exponential, or positive log‐normal, which means that they are certainly heavy‐tailed. However, log‐normal and truncated power law cannot be ruled out as potentially better suited for certain parts of the distributions in either of the graphs. The same result has been seen for many other real networks that do exhibit scale‐free behaviours [10].

### 3.2 Hierarchical Structure

The hierarchy of the graphs can be explored in several different ways. We analyse hubs, shortest path length, and betweenness centrality as well as clustering coefficient.

#### 3.2.1 Hubs

As previously seen in Figure 2, all graphs exhibit a small proportion of highly connected nodes. We follow [22] and select a threshold based on the average degree to determine which nodes should be considered hubs. First, we selected this threshold based on the total degree of the node, according to $\delta =100d_{\text{avg}}$, where $d_{\text{avg}}$ is the average total degree for the respective graphs. The resulting proportions of hubs, i.e. nodes with a high total degree, in the graphs with this definition are presented in Table 3. Unfortunately, this method resulted in hubs like ammonia, methyl iodide, BOC anhydride, methanol, and acetic anhydride. They are classified as reactants based on the atom‐mapping algorithm that identifies that they do contribute at least one heavy atom to the product. They are, however, not particularly interesting from a chemical perspective, as they are trivial, commercially available starting materials usually used for common transformations such as acetylation or BOC protection. We also acknowledge that they could be re‐classified based on some heuristic.

**TABLE 3:** Molecule hubs. Properties related to existing molecular hubs.

	ELN	USPTO	Reaxys
Average total degree	3.10	2.63	3.83
Hubs threshold	310.19	263.34	382.59
#Hubs (proportion)	136 (0.02%)	317 (0.03%)	4248 (0.04%)

**FIGURE 3:**
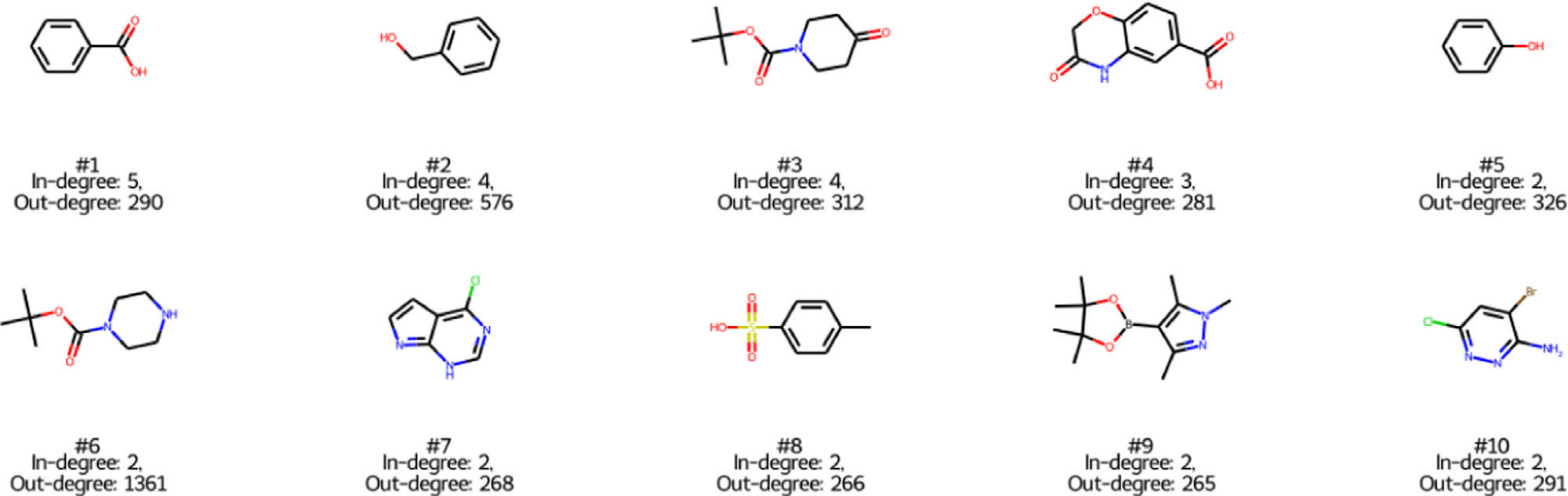
Molecular hubs. The top 10 ranked hubs based on the in degree from the ELN graph.

Instead, we continued the analysis by ranking the molecule nodes on the in‐degree of the nodes, and selected hubs that had an out‐degree of more than 100 of the average. To illustrate these molecules, the top 10 compounds with the highest in‐degrees from the three graphs are shown in Figure 3 from the ELN graph, as well as in Figures S1 and S2 for UPSTO and Reaxys respectively. Within these compounds, one can notice more advanced medicinal chemistry intermediates. For example, compound #3 can be used in sequential reductive amination and either amide or C–N cross‐coupling; compound #4 can be used in sequential O or N‐alkylation followed by amide coupling; compounds #6 and #10 can be used as intermediates in chemo‐ and regioselective C–N or C–C cross‐couplings. For ELN and USPTO, the hubs are various substituted ring systems, whereas Reaxys is a mix of ring systems and smaller compounds like carbon dioxide (#1), carbon monoxide (#6), and acetone (#9).

#### 3.2.2 Small‐World Behaviour

**FIGURE 4:**
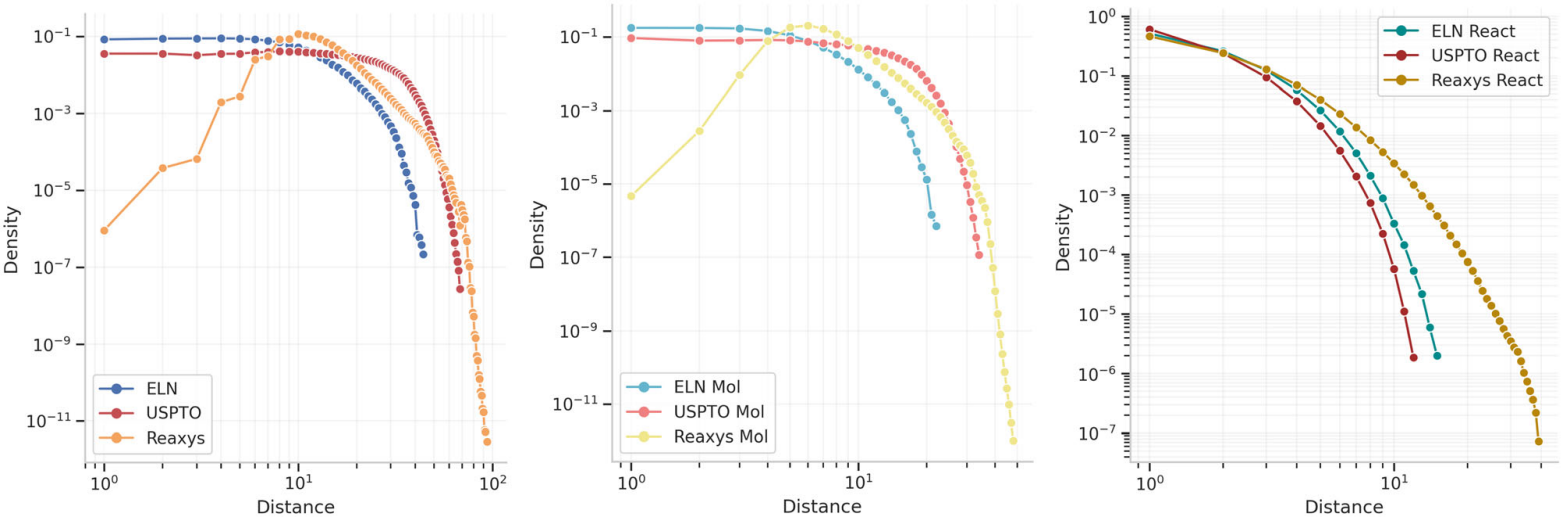
Distance distributions. (Left panel) when all nodes are considered, (Middle panel) when only the molecule nodes are considered in the middle, and (Right panel) when only the reaction nodes are considered in the right panel.

As all graphs include hubs, there is reason to believe that they might also exhibit small‐world properties, for which the average shortest path length should be small. Figure 4 shows the probability density function of shortest paths in each graph and Table S3 shows the average shortest path lengths for each graph and graph version. The molecular graph largely follows the bipartite graph's structure, and the reaction graph's structure is largely similar for all sources. However, for the bipartite or molecular graph, there are noticeable differences between the different sources. The ELN graph shows a steady distribution up to about a distance of 10, where the distribution drops rapidly. The Reaxys graph shows a steep increase up to about a distance of 10 before the distribution drops. Finally, the distribution for the USPTO graph stays steady up to about a distance of 100 before it drops.

#### 3.2.3 Measurements of Hierarchy

**FIGURE 5:**
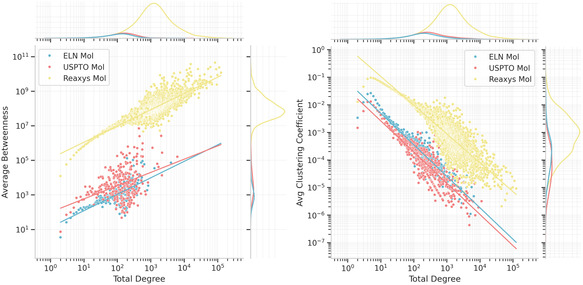
Hierarchical measurements. (Left) Average degree dependent betweenness: the margins show the distribution of the data and the lines represent a linear regression. (Right) Average degree dependent clustering: linear regressions of the respective data are shown as lines.

In addition to the presence of hubs and small‐world property, the hierarchy was measured in terms of average degree‐dependent betweenness; see Figure 5. We only show this analysis for the molecular graphs because the graphs for the reaction nodes show very little difference between data sources. One can observe that the betweenness centrality increases with the node degree. This is an indication of hierarchy in the graph, as it means that more connected nodes have more paths running through them. The trend is the same for all bipartite graphs as well as in the molecule and reaction versions.

The clustering coefficients for all bipartite graphs are exactly zero, as by design no triangles can exist in these graphs. Instead, the clustering coefficients were evaluated only on the molecule and reaction versions of the graphs. The global clustering coefficients of all monopartite graphs are presented in Table 4. Notably, the coefficients are all lower than the typical range of 0.01–0.5 found for many other graphs published in related work [11]. There appears to be very little difference between the clustering coefficients for the different graphs.

**TABLE 4:** Clustering coefficients. The average global clustering coefficients for each graph.

	ELN	USPTO	Reaxys
Molecule	0.00019_±4.62*e*−05_	0.00005_±1.08*e*−05_	0.00014_±2.28*e*−05_
Reaction	0.00010_±2.45*e*−05_	0.00039_±4.87*e*−05_	0.00005_±7.85*e*−05_

Regarding the local clustering of the graphs, it can be seen in Figure 5 that the average local clustering coefficient decreases with the node degree. This means that the nodes with high degrees are not part of clusters to the same extent as ones with fewer connections. As previously stated, some real‐world graphs have shown that the averaged degree‐dependent clustering coefficients scale according to $\bar{c}(k)\propto k^{-1}$ for hierarchical graphs [11]. Similar conclusions can be drawn for all molecule graphs in this analysis as their linear regression lines all have slopes close to −1.

### 3.3 Network Components and Properties

To identify the different components of interest, the first step was to determine whether the respective graphs had a core. To do so, the left panel in Figure S3 shows a distribution plot of the sizes of strong connected components (SCC) in each graph. The Reaxys graph has a clear such component according to the definition of a core, whereas USPTO can be seen to have a smaller core. However, ELN does not have a significantly larger SCC than the remaining ones. All graphs do, however, have a connected component (CC) that is significantly larger than any other; these can be referred to as the central components of the respective graphs, as seen in the right panel of Figure S3.

Each core was confirmed to be contained within the respective largest central components and therefore the periphery is defined as the relative complement of this central component and the core. Even for ELN, which did not have a clear core, the central component of the graph can still be taken to have a more central meaning than the remainder of the graph; hence, it is defined as this graph's periphery. All remaining nodes are considered to belong to islands and the resulting proportions of each structure are presented in Table 5. Additionally, the table presents the results for the molecule and reaction versions of the graphs as well as results found for the Beilstein database in [18].

**TABLE 5:** Structural components. The proportion of nodes in each bipartite graph that make up the core, periphery, and island components. The result for a precursor to Reaxys, the Beilstein database from [18], is also presented for comparison.

	ELN	USPTO	Reaxys	Beilstein
	1,192,831	2,159,007	24,972,473	∼5 900 000
Core	N/A	0.01%	6.94%	4%
Periphery	96.04%	88.14%	89.41%	78%
Islands	3.96%	11.85%	3.65%	18%

**FIGURE 6:**
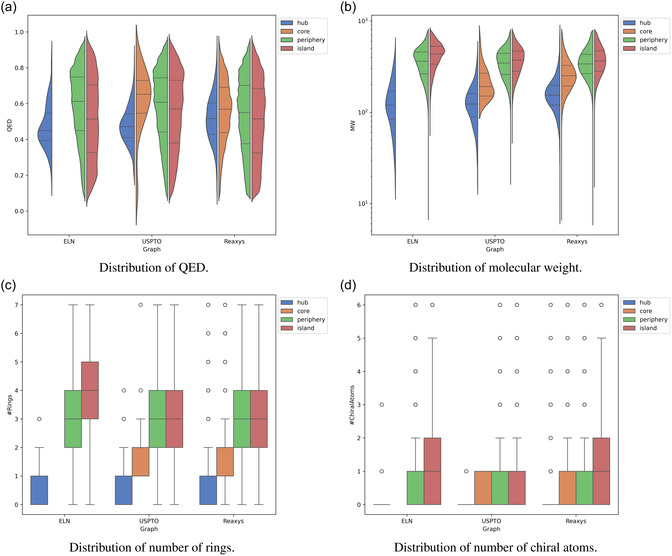
Distribution of molecular descriptors. Values that deviate from the mean with more than 2 times the standard deviation have been cropped from the plots.

Finally, we calculated properties related to molecular complexity and the QED score related to drug‐likeliness, averaged over each component as well as overall molecular hubs found in the respective graphs (see Figure 6 as well as Tables S4 and S5). The hypothesis from literature [6] is that hub and core molecules should be less complex and instead building block substances, i.e. smaller and diverse. Islands on the other hand are presumed to be made up of more complex, final drug compounds.

Molecular weight (MW) is one of the most simple indicators of molecular complexity. The MW is highest for molecules in islands for all graphs and becomes lower as you pass through the periphery into the core, with the lightest molecules on average being the hubs. Following the same trend is the number of heavy atoms, the number of rings, and the number of chiral centres, further corroborating the hypothesis. Only for the ELN graph do we observe an exception, where the average number of rings is higher in the periphery than in the islands. However, this property is less common for very small molecules where each carbon hybridization weighs heavier and as previously noted the hubs are significantly smaller based on the MW.

## 4 Discussion

In summary, we observe several differences between the three analysed reaction graphs. The largest graph derived from Reaxys is the most connected, as shown by the 1.49 ratio between the total number of edges and the total number of nodes. Furthermore, Reaxys shows the lowest power‐law parameter $\gamma_{\text{in}}$ of 5.19, the highest hierarchy, and the largest percentage of nodes that belong to the core. On the other hand, the graph derived from USPTO has the lowest general connectivity of 1.18, the highest $\gamma_{\text{in}}$ of 8.16, and a much lower percentage of nodes belong to the core. The graph derived from ELN often falls between these two extremes with a general connectivity of 1.30 and a $\gamma_{\text{in}}$ of 7.79. However, it does not have a definable core. Moreover, the hub molecules in the ELN and USPTO graphs are qualitatively similar and can be said to belong to small, organic building blocks, whereas some of the hub molecules in Reaxys are mostly inorganic compounds. The Reaxys hubs are on average heavier and have a higher QED score than the hubs in the USPTO and ELN graphs.

These differences might be explained to a large extent by the provenance of the data, i.e. the origin of the data. Reaxys is a comprehensive source of chemistry, encompassing both literature and patent data, whereas USPTO is confined to data from patents. ELN is a subset of reactions from a large pharmaceutical company, having a higher diversity of chemical moieties and a larger number of different chemical reactions. The scale‐free analysis shows that the knowledge structures within ELN and USPTO are more centralized around a few highly influential nodes compared to Reaxys. This expectation aligns with the nature of reaction data, especially in the context of pharmaceutical synthesis. For instance, when synthesizing new compounds, represented by newly added nodes of low connectivity, it is common to utilize more common reactions and building blocks, which are represented by highly connected nodes. As it was noted above, compounds #1, #3, #4, and #6 in Figure 3 are building blocks for the widely used amide coupling reaction, whereas compounds #7 and #10 are the building blocks for Pd‐catalysed cross‐coupling reactions. Following this and the fact that the ELN graph follows small‐world behaviour (the average shortest‐path is short), it can be suggested that hubs or hub‐like heavily connected nodes can be extracted and proposed for the experimentalists as good candidates to test less explored reactant partners with a higher expected reaction success rate. It can be speculated that additional filtering by the reaction type should be applied to make such a recommendation more accurate. In contrast, Reaxys exhibits a more evenly distributed connectivity network across its nodes, allowing for a broader spread of knowledge. This is further supported by the higher ratio of hubs in Reaxys, indicative of a more extensive exploration of chemistry across a broader and less restrictive part of the chemical space. In particular, one should probably pay attention and filter unrelated parts of the chemical space—, e.g., inorganic transformations—before using the Reaxys knowledge graph for medicinal chemistry synthesis planning or modelling. Furthermore, it is notable that highly connected nodes and hubs, particularly within the ELN and USPTO‐derived knowledge graphs, are characterized by lower QED scores (Figure 6a). This aligns with the assumption that these nodes are more likely to represent building blocks rather than final drug‐like compounds. Interestingly, this trend is not observed in the Reaxys graph.

Considering the ubiquitous use of USPTO data in the machine learning community, it is interesting to speculate on what the consequences of the differences observed herein are on the resulting models. If the purpose of the modelling is in the scope of drug discovery, it can be argued that USPTO represents some aspects of the chemical knowledge contained in an in‐house ELN reasonably well. However, there remain questions around reaction scope, something that we cannot readily analyse with graph theory. The knowledge contained in Reaxys naturally covers a larger reaction class variety, but as we have observed herein, it might not correspond to reactions of interest to drug discovery. Finally, we can likely conclude that the data in USPTO and ELN are prone to repetitions of similar syntheses, whereas the Reaxys data appears more diverse in terms of chemical reactions. It remains an open question whether the reaction knowledge graph can be used predictively in for instance yield models or building block selection. It is unclear if the graph contains knowledge beyond the information that can be extracted from individual reaction experiments.

## 5 Conclusion

We provide an extensive comparative study of the organic chemistry knowledge contained in three large sets of reaction data from literature, patents, and the pharmaceutical industry. As a result, we have shown that network theory provides valuable tools to analyse the structure of the knowledge graphs, but that there are also some limitations to this analysis. To a large extent, we found that the differences in the properties of the networks from the three sources can be rationalised by data provenance. Furthermore, the analysis shows that the distinct nature of the data in the three sources might have implications for predictive modelling using machine learning. As such, future research should carefully consider the source of the data used when training machine learning models for predictive tasks such as retrosynthesis and yield modelling.

## Conflicts of Interest

The authors declare no conflicts of interest.

## Supporting information

Supplementary Material

## Data Availability

The data that support the findings of this study are available from the corresponding author upon reasonable request.
