# Supplementary material for: Network Analysis of the Organic Chemistry in Patents, Literature, and Pharmaceutical Industry
Source: Mol Inform. 2025 Jul 18;44(7):e202500011. doi: 10.1002/minf.202500011 (PMC12273192; doi:10.1002/minf.202500011)
Supplement: Supplementary file 1 — Supplementary Material [file MINF-44-e202500011-s001.pdf]

Supporting Information for Network analysis of the organic chemistry in patents, literature, and pharmaceutical industry

Emma Svensson[1,2,*] **Emma Granqvist**[1,3,*] **Tomas Bastys**[1] **Christos Kannas**[1]

Mikhail Kabeshov[1] **Samuel Genheden**[1] **Ola Engkvist**[1,3] **Thierry Kogej**[1]

[1] **Molecular AI, Discovery Sciences, R&D**
AstraZeneca
Gothenburg, 431 83 Sweden

[2] **ELLIS Unit Linz & Institute for Machine Learning**
Johannes Kepler University Linz
Linz, 4040 Austria

[3] **Department of Computer Science and Engineering**
Chalmers University of Technology
Gothenburg, 412 96 Sweden

Correspondence to: thierry.kogej@astrazeneca.com

This document provides additional results related to the scale-free property, the hierarchical structure, and the network components as a complement to the analysis provided in the manuscript.

The Scale-free property

Table S1 presents the average degree of the bipartite and monopartite versions of the three graphs analyzed in this work. The statistical tests from comparing each degree distribution's fit between power law and other potential distributions are presented in Table S2.

[*]Equal contribution.

Table S1: **Average degree.** The average degree and standard deviation from the three versions of each graph included in the analysis.

	ELN	USPTO	Reaxys
**Bipartite**			
In	1.30±6.03e-04	1.18±4.60e-04	1.49±4.45e-04
Out	1.30±1.54e-02	1.18±1.39e-02	1.49±1.07e-02
Total	2.61±1.54e-02	2.37±1.39e-02	2.97±1.06e-02
**Molecule**			
In	1.55±1.11e-03	1.32±8.08e-04	1.91±9.11e-04
Out	1.55±2.84e-02	1.32±2.44e-02	1.91±2.10e-02
Total	3.10±2.84e-02	2.63±2.44e-02	3.83±2.15e-02
**Reaction**			
In	0.46±7.90e-04	0.35±5.64e-04	0.48±8.41e-04
Out	0.46±6.69e-04	0.35±4.93e-04	0.48±1.38e-04
Total	0.93±1.07e-03	0.70±7.74e-04	0.96±8.54e-04

Table S2: **Power law likelihood-ratio test.** Likelihood-ratio test results from comparing the power law fit to fits to other distributions. A positive R indicates that power law is a better fit for the data and a negative R indicates that the other distribution describes the data more accurately. The p-value is the significance of the R value. If $p > 0.1$ the direction of R is not reliable.

	Log-normal		Positive Log-normal		Truncated Power Law		Exponential		Stretched Exponential	
	R	p	R	p	R	p	R	p	R	p
**ELN**										
In	-3.16	0.2275	814.55	$\ll$ **0.1**	-3.12	**0.0125**	1129.19	$\ll$ **0.1**	102.31	$\ll$ **0.1**
Out	-34.27	$\ll$ **0.1**	1086.54	$\ll$ **0.1**	-25.82	$\ll$ **0.1**	30961.85	$\ll$ **0.1**	72.17	**0.0013**
Total	-6.32	**0.0413**	378.17	$\ll$ **0.1**	-5.24	**0.0012**	14137.31	$\ll$ **0.1**	64.84	**0.0001**
**USPTO**										
In	-13.86	$\ll$ **0.1**	170.62	$\ll$ **0.1**	$\ll 1$	0.998	441.05	$\ll$ **0.1**	840.55	$\ll$ **0.1**
Out	-5.96	**0.0555**	673.16	$\ll$ **0.1**	-5.03	**0.0015**	25806.9	$\ll$ **0.1**	2559.08	$\ll$ **0.1**
Total	-0.94	0.4658	195.69	$\ll$ **0.1**	-1.66	**0.0685**	9914.0	$\ll$ **0.1**	64.19	$\ll$ **0.1**
**Reaxys**										
In	-8646.08	$\ll$ **0.1**	287685.5	$\ll$ **0.1**	0.26	0.4671	952162.45	$\ll$ **0.1**	680110.25	$\ll$ **0.1**
Out	-5.69	$\ll$ **0.1**	13635.27	$\ll$ **0.1**	0.1	0.652	489006.62	$\ll$ **0.1**	35897.21	$\ll$ **0.1**
Total	-2.83	0.1554	89.22	$\ll$ **0.1**	-14.13	$\ll$ **0.1**	12277.41	$\ll$ **0.1**	56.63	$\ll$ **0.1**

Hierarchical structure

Figure S1 and S2 illustrates the top 10 molecular hubs found in USPTO and Reaxys compared to those found in ELN presented in Figure 2 in the main manuscript. The average shortest path lengths for the bipartite and monopartite versions of the three graphs are also presented in Table S3.

Table S3: **Average shortest path lengths.** Compared between the bipartite graph structure, the molecule-only graphs, and the reaction-only graphs.

	ELN	USPTO	Reaxys
Bipartite	7.24	15.44	13.14
Molecule	3.85	7.49	7.28
Reaction	1.90	1.65	2.28

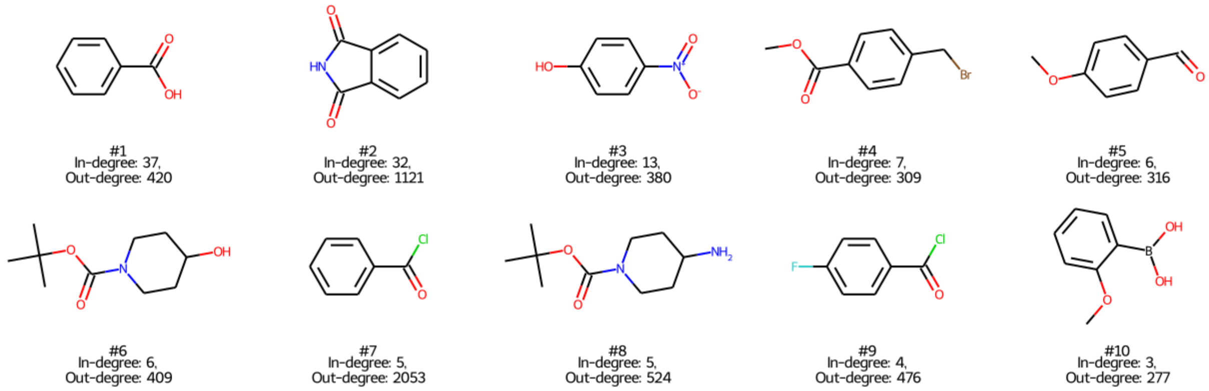

Figure S1: **Molecular hubs in USPTO, based on in degree.**

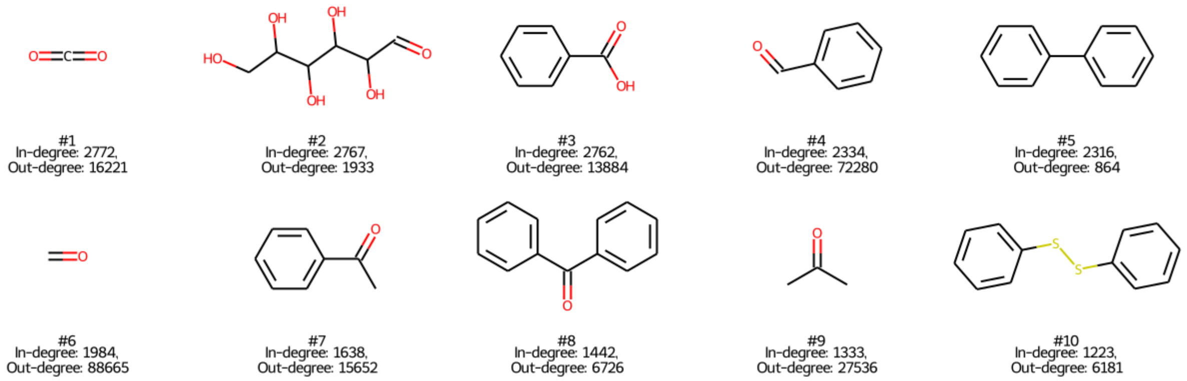

Figure S2: **Molecular hubs in Reaxys, based on in degree.**

Network components

Figure S3 presents histograms of component sizes from each graph, in terms of strongly connected components and connected components. Table S4 and S5 provide the full results of average molecular descriptors from network structures and node types respectively.

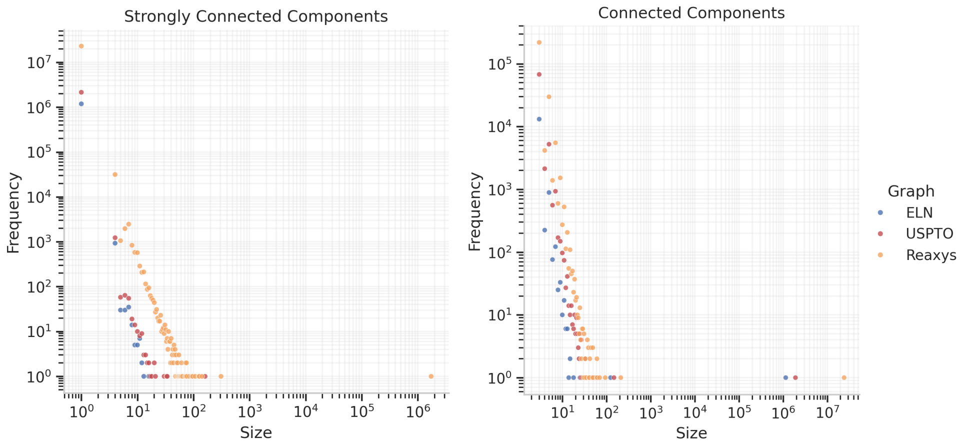

Figure S3: **Histogram of component sizes.** Right) Strongly Connected Components, and Left) Connected components in an undirected sense

Table S4: **Average molecular descriptors.** Molecular descriptors related to their complexity and drug-likeliness, averaged over all molecules in different sub-components of the two graphs. MW refers to the average molecular weight.

			Complexity ↑				Drug-Likeliness ↑
	#Molecules	MW	#HeavyAtoms	#Rings	#ChiralAtoms	Fsp3	QED
**ELN**							
Full	636,361	390.42$_{\pm 253.051}$	27.05$_{\pm 17.461}$	3.23$_{\pm 2.155}$	0.78$_{\pm 2.626}$	0.37$_{\pm 0.213}$	**0.58**$_{\pm 0.211}$
Island	31,128	478.27$_{\pm 321.604}$	**33.67**$_{\pm 22.023}$	**3.87**$_{\pm 2.180}$	**1.31**$_{\pm 3.070}$	0.40$_{\pm 0.185}$	0.49$_{\pm 0.241}$
Periphery	605,233	385.90$_{\pm 248.176}$	26.71$_{\pm 17.125}$	3.20$_{\pm 2.149}$	0.75$_{\pm 2.598}$	0.37$_{\pm 0.214}$	**0.58**$_{\pm 0.208}$
Hubs	192	134.17$_{\pm 76.221}$	7.92$_{\pm 5.118}$	0.60$_{\pm 0.803}$	0.05$_{\pm 0.327}$	**0.48**$_{\pm 0.414}$	0.47$_{\pm 0.119}$
**USPTO**							
Full	1,224,059	363.42$_{\pm 133.785}$	25.13$_{\pm 9.702}$	2.91$_{\pm 1.478}$	0.61$_{\pm 1.155}$	0.35$_{\pm 0.215}$	0.57$_{\pm 0.205}$
Island	168,723	388.92$_{\pm 146.199}$	**27.25**$_{\pm 10.483}$	**3.09**$_{\pm 1.505}$	0.93$_{\pm 1.552}$	0.37$_{\pm 0.214}$	0.54$_{\pm 0.221}$
Periphery	1,055,287	359.34$_{\pm 131.229}$	24.79$_{\pm 9.528}$	2.88$_{\pm 1.472}$	0.56$_{\pm 1.069}$	0.35$_{\pm 0.215}$	0.58$_{\pm 0.202}$
Core	49	245.61$_{\pm 155.027}$	17.59$_{\pm 11.277}$	1.63$_{\pm 1.305}$	**0.98**$_{\pm 2.124}$	0.23$_{\pm 0.204}$	**0.62**$_{\pm 0.176}$
Hubs	435	131.88$_{\pm 62.806}$	7.66$_{\pm 3.859}$	0.60$_{\pm 0.705}$	0.02$_{\pm 0.126}$	**0.47**$_{\pm 0.418}$	0.48$_{\pm 0.104}$
**Reaxys**							
Full	11,851,702	380.76$_{\pm 197.160}$	26.33$_{\pm 14.035}$	2.93$_{\pm 2.134}$	1.35$_{\pm 2.643}$	0.39$_{\pm 0.254}$	0.52$_{\pm 0.222}$
Island	588,857	439.29$_{\pm 274.909}$	**30.74**$_{\pm 19.352}$	**3.48**$_{\pm 2.638}$	**1.93**$_{\pm 3.678}$	0.41$_{\pm 0.253}$	0.48$_{\pm 0.239}$
Periphery	10,877,693	381.16$_{\pm 192.515}$	26.35$_{\pm 13.710}$	2.93$_{\pm 2.106}$	1.32$_{\pm 2.586}$	0.38$_{\pm 0.252}$	0.52$_{\pm 0.222}$
Core	385,152	279.80$_{\pm 135.819}$	18.80$_{\pm 9.688}$	1.87$_{\pm 1.658}$	1.21$_{\pm 2.174}$	**0.44**$_{\pm 0.301}$	**0.55**$_{\pm 0.183}$
Hubs	5,421	169.79$_{\pm 86.253}$	10.81$_{\pm 5.788}$	1.01$_{\pm 1.172}$	0.22$_{\pm 0.998}$	0.38$_{\pm 0.368}$	0.52$_{\pm 0.131}$

Table S5: **Average molecular descriptors.** Molecular descriptors related to their complexity and drug-likeliness, averaged over all molecules in different node types of the two graphs: *initial*, *intermediate*, and *terminal* nodes. MW refers to the average molecular weight.

			Complexity ↑				Drug-Likeliness ↑
	#Molecules	MW	#HeavyAtoms	#Rings	#ChiralAtoms	Fsp3	QED
**ELN**							
Full	636,361	$390.42_{\pm 253.05}$	$27.05_{\pm 17.46}$	$3.23_{\pm 2.16}$	$0.78_{\pm 2.63}$	$0.37_{\pm 0.21}$	$0.58_{\pm 0.21}$
Primary	105,676	$322.65_{\pm 263.80}$	$21.93_{\pm 18.20}$	$2.47_{\pm 2.05}$	$0.76_{\pm 2.62}$	$0.40_{\pm 2.66}$	$0.59_{\pm 0.21}$
Intermediate	240,065	$364.25_{\pm 170.22}$	$24.91_{\pm 12.12}$	$2.85_{\pm 1.64}$	$0.62_{\pm 1.51}$	$0.38_{\pm 0.215}$	$0.60_{\pm 0.21}$
Terminal	290,620	$436.68_{\pm 294.27}$	$30.68_{\pm 19.98}$	$3.83_{\pm 2.39}$	$0.92_{\pm 0.21}$	$0.36_{\pm 0.21}$	$0.55_{\pm 0.21}$
**USPTO**							
Full	1,224,059	$363.42_{\pm 133.79}$	$25.13_{\pm 9.70}$	$2.91_{\pm 1.48}$	$0.61_{\pm 1.16}$	$0.35_{\pm 0.22}$	$0.57_{\pm 0.21}$
Primary	314570	$331.1_{\pm 137.37}$	$22.66_{\pm 9.87}$	$2.56_{\pm 1.43}$	$0.64_{\pm 1.20}$	$0.37_{\pm 0.24}$	$0.59_{\pm 0.20}$
Intermediate	307,776	$338.94_{\pm 126.63}$	$23.14_{\pm 9.18}$	$2.59_{\pm 1.35}$	$0.51_{\pm 1.02}$	$0.34_{\pm 0.22}$	$0.60_{\pm 0.20}$
Terminal	601,713	$392.82_{\pm 128.94}$	$27.43_{\pm 9.32}$	$3.25_{\pm 1.49}$	$0.65_{\pm 1.17}$	$0.34_{\pm 0.20}$	$0.55_{\pm 0.21}$
**Reaxys**							
Full	11,851,702	$380.76_{\pm 197.16}$	$26.33_{\pm 14.04}$	$2.93_{\pm 2.13}$	$1.35_{\pm 2.64}$	$0.39_{\pm 0.25}$	$0.52_{\pm 0.22}$
Primary	1,266,902	$388.47_{\pm 240.13}$	$26.64_{\pm 16.91}$	$2.86_{\pm 2.36}$	$1.50_{\pm 3.31}$	$0.41_{\pm 0.27}$	$0.50_{\pm 0.23}$
Intermediate	4,388,223	$367.58_{\pm 194.40}$	$25.27_{\pm 13.90}$	$2.64_{\pm 1.90}$	$1.42_{\pm 2.64}$	$0.41_{\pm 0.26}$	$0.52_{\pm 0.22}$
Terminal	6,196,577	$388.5_{\pm 188.65}$	$27.00_{\pm 13.43}$	$3.14_{\pm 2.22}$	$1.27_{\pm 2.48}$	$0.37_{\pm 0.25}$	$0.51_{\pm 0.22}$
